# Two decades of a protooncogene TBL1XR1: from a transcription modulator to cancer therapeutic target

**DOI:** 10.3389/fonc.2024.1309687

**Published:** 2024-01-29

**Authors:** Ruijuan Du, Kai Li, KeLei Guo, Zhiguo Chen, Xulin Zhao, Li Han, Hua Bian

**Affiliations:** ^1^ Zhang Zhongjing School of Chinese Medicine, Nanyang Institute of Technology, Nanyang, Henan, China; ^2^ Henan Key Laboratory of Zhang Zhongjing Formulae and Herbs for Immunoregulation, Nanyang Institute of Technology, Nanyang, Henan, China; ^3^ Oncology Department, Nanyang First People’s Hospital, Nan Yang, Henan, China

**Keywords:** TBL1XR1, oncogene, upstream regulators, signaling pathways, therapeutic target

## Abstract

Transducin beta-like 1X-related protein 1 (TBL1XR1) was discovered two decades ago and was implicated as part of the nuclear transcription corepressor complex. Over the past 20 years, the emerging oncogenic function of TBL1XR1 in cancer development has been discovered. Recent studies have highlighted that the genetic aberrations of TBL1XR1 in cancers, especially in hematologic tumors, are closely associated with tumorigenesis. In solid tumors, TBL1XR1 is proposed to be a promising prognostic biomarker due to the correlation between abnormal expression and clinicopathological parameters. Post-transcriptional and post-translational modification are responsible for the expression and function of TBL1XR1 in cancer. TBL1XR1 exerts its functional role in various processes that involves cell cycle and apoptosis, cell proliferation, resistance to chemotherapy and radiotherapy, cell migration and invasion, stemness and angiogenesis. Multitude of cancer-related signaling cascades like Wnt-β-catenin, PI3K/AKT, ERK, VEGF, NF-κB, STAT3 and gonadal hormone signaling pathways are tightly modulated by TBL1XR1. This review provided a comprehensive overview of TBL1XR1 in tumorigenesis, shedding new light on TBL1XR1 as a promising diagnostic biomarker and druggable target in cancer.

## 1 Introduction

**Figure 1:**
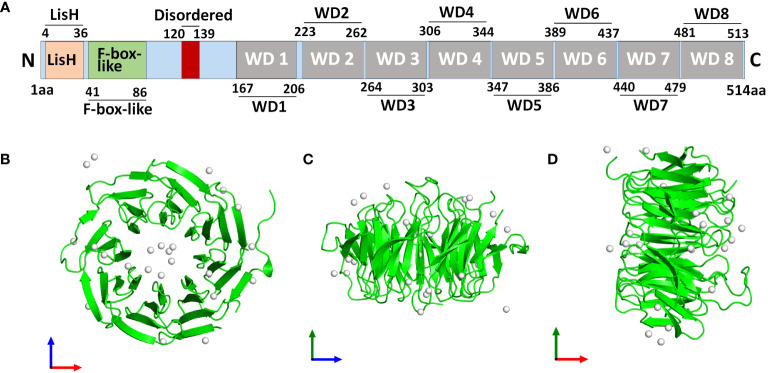
Structure of human TBL1XR1 protein. The protein structure was obtained from Protein Data Bank (https://www.rcsb.org/structure/4LG9). **(A)** The domain structure of human TBL1XR1. Monomeric assembly 1 of PDB entry 4lg9 was coloured by chemically distinct molecules and viewed from the front **(B)**, the side **(C)** and the top **(D)**.

The WD40 repeat-containing gene family includes the protein known as TBLR1, also known as transducin beta-like 1X-related protein 1 (TBL1XR1). The Trp-Asp dipeptide, referred to as the “WD” and typically ends the repeat, is a part of the roughly 40 amino acid long WD40 repeat domain (1). A total length of 514 amino acid protein with a molecular mass of 55.5 kDa is encoded by human TBL1XR1 gene. TBL1XR1 is composed of a C-terminal WD40 repeat domain whose function is crucial for interactions between both protein-DNA and protein-protein, an N-terminal LisH domain for chromatin distribution and hetero- and homodimerization and an F-Box domain for E3 ligase enrollment (**Figure 1A**) (2, 3). TBL1XR1’s first WD repeat is necessary for it to establish a connection to the RD4 domain of the NCoR (4). WD40 domains frequently fold into four- to eight-bladed -propellers that form a funnel-like form within proteins (**Figures 1B–D**) (5).

**Figure 2:**
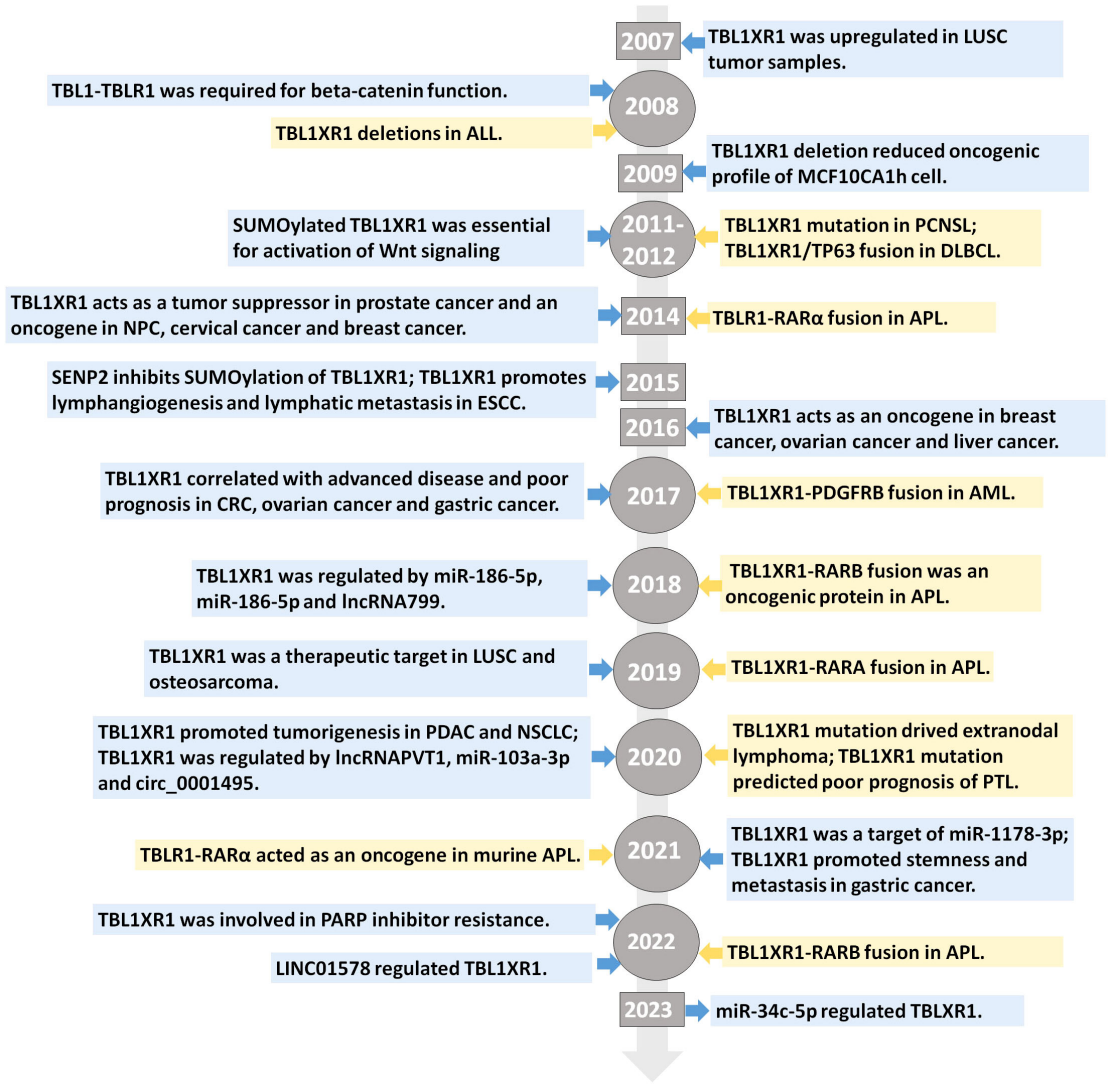
Evolution tree of TBL1XR1. The highlight of discovery of TBL1XR1 and its significance in the field of cancer research.

By serving as a scaffold protein that can be a component of the nuclear corepressor/coactivator complex, WD40 domains influence cellular processes by controlling the transcription of genes (1). In 1999, a member of the TBL1X gene family that was either completely or partially deleted in patients with Xp22.3 terminal deletions was discovered (6). TBL1XR1 was then identified in the year 2003 as the heterogenous 55 kDa protein with TBL1X, both of which participated in the formation of transcriptional suppressor complexes that also contain N-CoR, SMRT, as well as histone deacetylase 3 (HDAC 3) (4, 7, 8). TBL1XR1 is a gene with a high degree of similarity to the TBL1X protein that is found on chromosome 3 at 3q26. Additionally, SMRT, N-CoR, and WD40 repeats can interact with TBL1X and TBL1XR1 at their N terminals (4, 8). Expect for interacting with SMAR/N-CoR corepressor complex, to activate transcription, β-catenin and TBL1-TBLR1 formed a large complex at the site of Wnt target-gene promoter (9). TBL1XR1 protein’s structural stability was compromised by the Phe10Leu mutation, which also changed Wnt signaling activity (10). Additionally, it has been suggested that TBL1XR1 functions as an E3 ubiquitin ligase, attracting the 19S proteasome and UbcH5 ubiquitin conjugating enzymes before replacing corepressors for coactivators in a ligand-dependent manner (11). Two decades have passed since the discovery of TBL1XR1 and in unraveling its cellular functions. In early years, researches frequently reported the variants of TBL1XR1 in neurodevelopmental disorders, such as Pierpont syndrome (12–14), autism spectrum disorders (15, 16), West syndrome (17, 18) and intellectual disability (19). Later it emerged as a potential biomarker of poor prognosis and tumorigenesis for different types of cancers. We made a systematic evolutionary tree of TBL1XR1 that showed the explorations made so far with respect to its relevant researches in cancers (**Figure 2**). We also revealed a comprehensive overview of the transcript variants of TBL1XR1 and its expression and clinical significance in cancers. The oncogenic role and regulatory mechanisms of TBL1XR1 is also discussed. When taken as a whole, targeting TBL1XR1 is a novel and interesting approach to the detection and treatment of cancer.

## 2 Genetic aberrations of TBL1XR1

Genetic changes of TBL1XR1 such as gene variations and gene arrangement were present in cancers especially in hematologic tumors including lymphoma and leukemia. 15% of acute lymphoblastic leukemia (ALL) patients with the ETV6-RUNX1 fusion had TBL1XR1 deletions, which made SMRT/N-CoR less effective at governing gene expression appropriately (20). TBL1XR1 somatic mutations were found in 14% of primary central nervous system lymphoma (PCNSL) cases (21). The amino acid changes were D421H, S419F, S419T, F541S, V445V in these cases (21). Re-wiring of germinal center B cell (GCB) to drive lymphomagenesis was caused by TBL1XR1 mutations in conditional TBL1XR1-D370Y mice (22). Transcriptional reprogramming of pre-memory and cell-fate bias are caused by TBL1XR1 mutants coaxing SMRT/HDAC3 repressor complexes to bind the transcription factor BACH2 instead of BCL6 (22). Another study found that patients with TBL1XR1 mutations in primary testicular lymphoma (PTL) had worse overall survival and were more likely to have more tumor infiltration (23).

Fusion gene transcripts, which largely results from cryptic chromosomal rearrangement, are hallmarks of hematologic malignancy. The newly identified TBL1XR1/TP63 gene fusion was discovered using transcriptome sequencing in diffuse large B-cell lymphoma (DLBCL) (24). Both of retinoic acid receptor alpha (RARA) and another family gene retinoic acid receptor beta (RARB) are strongly important members of the nuclear receptor superfamily. In acute promyelocytic leukemia (APL), TBLR1-RARA (25, 26) and TBLR1-RARB (27, 28) gene fusion were identified. TBLR1-RARA fusion proteins act as a transcriptional activator in APL by self-assembling into homodimers (25). Under the therapy with all-trans retinoic acid (ATRA), TBLR1-RARA caused transcriptional corepressors to dissociate and degrade, which in turn transactivated the transcription of RARA target genes and subsequently provoked cell differentiation (25). In a mouse model with TBLR1-RARA expression, TBLR1-RARA performed as an oncogene to trigger APL-like disease and HDAC inhibitors rather than ATRA or As2O3 conferred survival advantage against TBLR1-RARA expression mice (25). One TBL1XR1-RARB fusion-positive APL patient reacted well to traditional chemotherapy but was largely resistant to ATRA and arsenic trioxide treatment (28). Another report in an acute myeloid leukemia (AML) patient identified a novel fusion TBL1XR1-PDGFRB and this fusion may be sensitive to dasatinib (29).

## 3 Subcellular location of TBL1XR1 in cancer

TBL1XR1 was primarily restricted in the nucleus in original CRC tissues and metastases tumor of liver, exhibiting little positive staining in the cytoplasm (30, 31). The majority of TBL1XR1 was found in the nucleus of tumor cells in ovarian cancer (32), ESCC (33), NPC (34), gastric cancer (35) in patient tissues. In breast and ovarian cancer, TBL1XR1 was primarily found in the nucleus and in small amounts in the cytoplasm (36). Significantly higher nuclear TBL1XR1 expression instead of cytoplasm TBL1XR1 was observed in the breast cancer malignant glands compared to the adjacent benign breast glands (36). Comparing each type of ovarian epithelial carcinomas to relatively healthy fallopian tubes, TBL1XR1 was expressed at a relatively upregulated level in both the nucleus and cytoplasm of cancer cells (36). In osteosarcoma, TBL1XR1 expression was primarily found in the nucleus and only rarely in the cytoplasm (37). TBL1XR1’s cytoplasmic staining did not notably correlate with clinicopathologic factors or disease prognosis of osteosarcoma patients (37). TBL1XR1 was mostly discovered in the nucleus of benign prostate cell line and benign prostatic glands, whereas it was found in both the cytoplasm and nucleus in cancer cells and malignant glands (38). Functionally, TBL1XR1 translocated to the nucleus and induced a growth arrest upon serum starvation (38). TBL1XR1 is typically found in the nucleus of cancer cells, but in hepatocellular carcinoma tumor cells, TBL1XR1 staining was primarily distributed in the cytoplasm (39).

## 4 Expression and clinical significance of TBL1XR1 in cancer

Increasing research has shown that TBL1XR1 overexpression in cancer is closely connected to clinical traits. Subsequently, the TBL1XR1 expression profile and clinical importance in cancer according to the reported literature was elaborated in the following part and summarized in **Table 1**.

**Table 1:** TBL1XR1 expression in various cancers.

Cancer types	Expression in tumor tissues	Clinical significance	Methods	References
Lung cancer	Upregulation	NA	qRT-PCR, western blot	(40)
	Upregulation	NA	qRT-PCR, western blot	(41)
	Upregulation	TBL1XR1 expression was related with poor prognosis.	qRT-PCR, western blot	(42)
	Upregulation	TBL1XR1 expression was related with poor prognosis.	GEO, PROGgene	(43)
Breast cancer	Upregulation	TBL1XR1 was correlated with clinical stage, the tumor classification , node classification, metastasis classification and histological grade , as well as with the expression of c-erbB2 and Ki-67. Higher TBL1XR1 expression was an independent prognostic indicator for the survival time.	qRT-PCR, western blot, IHC	(44)
	Upregulation	TBL1XR1 nuclear expression was related with AR and PR expression.	IHC	(36)
Hepatocellular carcinoma	Upregulation	TBL1XR1 was related to serum alpha fetoprotein , clinical stage, maximum size of tumors, tumor embolus , histological grade and predicted patient poor survival.	qRT-PCR, western blot, IHC	(39)
	Upregulation	NA	qRT-PCR	(45)
Gastric cancer	Upregulation	TBL1XR1 was associated with clinical stage, lymph node metastasis, number of lymph node and poor prognosis.	IHC	(46)
	Upregulation	TBL1XR1 levels correlated with local tumour invasion, late tumor, lymph node, TNM stage and poor prognosis.	IHC	(35)
Prostate cancer	Gene gains/amplifications	TBL1XR1 expression was related with higher Gleason grade and stage.	cBioportal database	(47)
	Downregulation	NA	IHC	(38)
Ovarian cancer	Upregulation	TBL1XR1 expression was related with FIGO stage, lymph node metastasis and overall survival.	IHC, qRT-PCR, western blot	(32)
	Upregulation	NA	IHC	(36)
Osteosarcoma	Upregulation	NA	qRT-PCR, western blot	(48)
	Upregulation	TBL1XR1 expression was associated with present metastasis, advanced Enneking stage and patient prognosis.	IHC, western blot	(37)
Colorectal cancer	Upregulation	TBL1XR1 was correlated with liver metastases and was an independent prognostic factor.	IHC	(30)
Esophageal squamous cell carcinoma	Upregulation	TBL1XR1 expression correlated with ESCC stage and patient survival, and was an independent prognostic factor for patient outcome.	IHC, qRT-PCR, western blot	(33)
Cervical cancer	Upregulation	TBL1XR1 correlated with FIGO stage, stromal invasion, lymphovascular space invasion, pelvic lymph node metastasis, parametrial infiltration, high-risk HPV infection, postoperative adjuvant chemotherapy, recurrence and survival.	IHC, qRT-PCR, western blot	(49)
Pancreatic ductal adenocarcinoma	Upregulation	TBL1XR1 was associated with TNM stage and was a significant independent prognostic factor.	IHC	(50)
Nasopharyngeal carcinoma	Upregulation	TBL1XR1 was correlated with clinical stage, T classification, N classification and patient survival and was a significant independent prognostic factor.	IHC, qRT-PCR, western blot	(34)

### 4.1 Lung cancer

At the very beginning, TBL1XR1 was reported to be upregulated in 75.0% (21/28) of LUSC tumour tissues at mRNA level and in 53.3% (8/15) of LUSC tumor samples at protein levels (40). Moreover, TBL1XR1 expression increased in a total of five lung cancer cell lines compared with one normal bronchial epithelial cell line (40). Another study showed significantly higher TBL1XR1 mRNA levels in 30 paired NSCLC tissues and higher protein levels in 3 matched NSCLC tissues (41). TBL1XR1 expression in serum of NSCLC patients was also elevated compared with normal participants (41). In addition, both TBL1XR1 mRNA and protein expression in paired NSCLC tissues was measured and TBL1XR1 was significantly upregulated in tumor tissues and predicted poor prognosis in NSCLC (42). TBL1XR1 was also found to be one of the genes with differential expression in SCC and COPD cases, and it controls the pathogenesis of LUSC triggered by COPD (43). When stratified with clinicopathological factors, TBL1XR1 was related with shorter overall survival in LUSC patients constructed from a PROGgene clinical microarray dataset (43). These findings pointed out that TBL1XR1 might be a potential lung cancer therapeutic target, especially for NSCLC.

### 4.2 Breast cancer

TBL1XR1 was found to be overexpressed in breast cancer tissues and cells when compared to healthy controls. Immunohistochemical (IHC) evaluation of archived paraffin-embedded breast cancer samples revealed that nearly half of them were tested positive for TBL1XR1 (44). Furthermore, TBL1XR1 expression was also linked to c-erbB2 and Ki-67 expression levels, as well as clinical stage, tumor categorization, classification of nodes, metastasis categorization, and histological grade (44). According to multivariate analysis, TBLR1 expression may serve as an independent prognostic factor for patients’ survival of breast cancer (44). According to Xinyu Wu et al., there was no discernible distinctions in the level of TBL1XR1 cytoplasmic expression between malignant and benign breast glands, but there was a significant distinctions in the nucleus expression of TBL1XR1 in malignant glands compared to the nearby benign breast glands (36). Additionally, androgen receptor (AR) and progesterone receptor (PR) positive cases had higher levels of TBL1XR1 expression in the nuclear of cancer cells (36).

### 4.3 Hepatocellular carcinoma

Xuejun Kuang et al. demonstrated that TBL1XR1 expression levels were elevated in LIHC cell lines and tissues (39). Furthermore, increased serum alpha fetoprotein levels, more tumor emboli, advanced clinical phases, larger maximum tumor sizes, and an aggressive histological grade were all associated with TBL1XR1 upregulation in LIHC (39). The disease-free as well as overall survival times were typically shorter in patients with elevated TBL1XR1 expression (39). Another investigation found that LIHC tissues had significantly elevated TBL1XR1 expression compared to the nearby normal tissues (45). Collectively, TBL1XR1 might serve as a potential prognostic marker and therapeutic target for LIHC.

### 4.4 Gastric cancer

Upregulated TBL1XR1 in gastric cancer has been linked to a progressed clinical phase, growing lymph node numbers and metastasis of lymph node (46). Additionally, patients who expressed TBL1XR1 highly possessed poorer prognosis (46). Another study reported that 61.2% gastric cancer tissues and 29.8% nearby normal tissues can be detected for TBL1XR1 expression (35). The total amount of cells with positive TBL1XR1 expression in gastric tumor tissues was noticeably higher than in nearby normal tissues (35). Furthermore, local invasion and the progressed TNM stage were strongly linked with the levels of the TBL1XR1 protein (35). Patients who had elevated TBL1XR1 expression possessed shorter overall survival, according to a survival analysis (35). In gastric cancer cell lines, TBL1XR1 expression was also evaluated when compared to the normal gastric epithelial cell line (35). These studies suggested that TBL1XR1 has been elevated in gastric cancer and may be crucial for the development of the disease.

### 4.5 Prostate cancer

According to the TCGA database, one study found that TBL1XR1 gene gains or amplifications were more prevalent in aggressively malignant subtypes of prostate cancer than in primary cohorts (47). However, in another study, nuclear TBL1XR1 expression was markedly reduced in both prostate cancer cells and human tumor samples compared with benign prostate cells or adjacent benign prostatic glands (38). The oncogenic or tumor suppressive role of TBL1XR1 in prostate cancer was to be further investigated.

### 4.6 Ovarian cancer

TBL1XR1 levels of expression increased in serous epithelial ovarian cancer tissues than in normal ovarian tissues, and elevated degrees of TBL1XR1 were indicative of FIGO phase and lymph node metastasis (32). Additionally, increased TBL1XR1 levels were linked with a poor prognosis for ovarian cancer patients (32). When comparing every types of ovarian epithelial carcinomas to healthy fallopian tubes, another study discovered an elevated levels of TBL1XR1 throughout both the nucleus and cytoplasm (36). In general, TBL1XR1 might serve as a new target for treatment and a diagnostic tool for ovarian cancer.

### 4.7 Osteosarcoma

Osteosarcoma samples had significantly higher levels of TBL1XR1 expression than the paired adjacent non-tumor tissues (48). Likewise, comparatively to the normal human osteoplastic cell line, TBL1XR1 levels was elevated among all osteosarcoma cell lines both at the protein and mRNA levels (48). Another study also demonstrated the upregulation of TBL1XR1 in osteosarcoma tumor tissues and TBL1XR1 expression was positively linked to present metastasis and advanced Enneking stage (37). Additionally, TBL1XR1 expression was identified as an independent prognostic factor for osteosarcoma patients (37). The results of the two investigations suggested that TBL1XR1 may be crucial for the growth of osteosarcoma.

### 4.8 Colorectal cancer

TBL1XR1 was found to have prominent expression levels in the nuclear of tumor cells in 57.4% of colorectal cancer (CRC) tissues and in 72.3% of liver metastases (30). Abnormal expression of TBL1XR1 in CRC cancer tissues was strongly linked with high proportions of metastases towards liver and was an independent prognostic factor for tumor recurrence (30). Significant TBL1XR1 expression is additionally linked with poor patient disease-free survival in TNM stage I to stage III (30).

### 4.9 Other cancers

TBL1XR1 expression has been displayed to be substantially increased in esophageal squamous cell carcinoma (ESCC) tumor tissues, which was highly associated with a more progressed ESCC disease stage and a worse patient survival, and recognized as an independent prognostic factor (33). Moreover, both cervical cancer cell lines and cervical cancer tissues showed upregulation of TBL1XR1 (49). Strong correlations between elevated TBL1XR1 levels and FIGO phase, lymphovascular space invasion, pelvic lymph node dissemination, stromal spread, parametrial infiltration, high-risk human papillomavirus (HPV) infection, adjuvant chemotherapy, recurrence, as well as patient survival have been observed (49). Moreover, TBL1XR1 was identified as an independent prognostic marker for the outcome of cervical cancer patients (49). Patients with low TBL1XR1 expression exhibited a more prolonged overall time than those with high TBL1XR1 expression in pancreatic ductal adenocarcinoma (PDAC), and excessive expression of TBL1XR1 was linked with TNM phase (50). More importantly, TBL1XR1 was considered as a significant independent prognostic factor in PDAC (50). Additionally, TBL1XR1 expression was found to be raised in nasopharyngeal carcinoma (NPC) cell lines, detectable in 89.52% tumor tissues, and highly expressed in 49.52% of the NPC tumor tissues (34). The clinical phase of NPC, the T stage, the N stage, as well as patient survival time were all correlated with TBL1XR1 levels of expression (34).

## 5 Oncogenic functions of TBL1XR1 in cancers

As TBL1XR1 was abnormally expressed in cancers and related with advanced diseases and predicted poor prognosis, TBL1XR1 must have participated in various processes of cancer development and further progression. This section focuses on the functions of TBL1XR1 in carcinogenesis and provides functional validation for its oncogenic feature.

### 5.1 Role of TBL1XR1 in cell proliferation and tumor growth

Cell proliferation is extremely necessary for normal biological processes of several adult tissues, embryogenesis, growth, and tumorigenesis. The effects of TBL1XR1 on controlling cancer cell proliferation have recently come into focus in new investigations. In PDAC cells, TBL1XR1 knockdown inhibited cell growth and colony formation *in vitro* and decreased tumor expansion in mice models (50). In NSCLC cell lines, TBL1XR1 deletion dramatically reduced cell proliferation, whereas TBL1XR1 amplification increased cell proliferation according to data collected by the MTT assay (41). In prostate cell lines, absence of TBLR1 expression resulted in a sharp decline in cell proliferation (38). In breast cancer tissues, TBL1XR1 expression was related with proliferation marker Ki67, indicating its important role in cell proliferation (44). In breast cancer, gastric cancer and LUSC cells, ectopic overexpression of TBL1XR1 increased cell proliferation and TBL1XR1 silencing inhibited the proliferative capacity (35, 44, 51). TBL1XR1 also promoted breast cancer and gastric cancer cell colony formation and tumor expansion in a xenograft model (35, 44).

### 5.2 Role of TBL1XR1 in cell cycle and cell apoptosis

TBL1XR1 downregulation also caused PDAC cell cycle arrest at the G0/G1 phase, in part via affecting the expression levels of genes that control the cell cycle, such as CDK2, CDC25A, and cyclin D1 (50). TBL1XR1 accelerated cell cycle progression as evidenced by a rise in S and G2/M phase cells and a reduction in G0/G1 phase cells (41). TBL1XR1 knockdown PDAC cells had a clearly greater proportion of apoptotic cells, which also had increased Bax and Bad expression and lowered Bcl-2 expression (50).

### 5.3 Role of TBL1XR1 in chemoresistance and radioresistance

More than 90% of cancer patients’ deaths are attributed to the resistance to chemotherapeutic agents and it is urgent to clarify the molecular mechanism responsible for chemoresistance (52). Both NSCLC and NPC cells were more sensitive to cisplatin after TBL1XR1 knockout and more resistant to cisplatin treatment after TBL1XR1 overexpression (34, 41). Moreover, after cisplatin treatment, growth of xenografted tumors with TBL1XR1 knockdown was remarkably inhibited while TBL1XR1 overexpression maintained the tumor growth (34). Another research in gastric cancer also indicated that TBL1XR1 knockdown reversed resistance to cisplatin *in vitro* and *in vivo (* 46). Gastric tissues and cells that were resistant to cisplatin displayed an evident boost in TBL1XR1 expression (53). CRISPR screen experiments identified TBL1XR1 as a candidate gene associated with PARP inhibitor resistance in prostate cancer cells (54). A greater sensitivity to the PARP inhibitor olaparib was caused by TBL1XR1 deletion (54). Additionally, in both 2D and 3D culture, the olaparib-resistant cells with TBL1XR1 deletion were more susceptible to olaparib treatment (54). In terms of mechanism in prostate cancer cells, PARP1 plays an important role in the susceptibility of TBL1XR1 deletion cells to PARP inhibitors (54). Suppression of the NCoR complex or therapy with the HDAC inhibitor rescues responsiveness to glucocorticoids (55). TBL1XR1 deletion may be a new factor of glucocorticoid resistance in ALL (55). Radiation therapy resistance is still a significant clinical issue that negatively affects the prognosis of cancer patients. TBL1XR1 was associated with radioresistance in the same way as chemoresistance. According to a publication, in the radiation-resistant NSCLC cell lines, TBL1XR1 expression was obviously upregulated and increased TBL1XR1 expression enhanced the biological malignancy of these cells (42).

### 5.4 Role of TBL1XR1 in tumor metastasis

The researches illustrating TBL1XR1’s function in foretelling malignant phenotypes, such as cancer metastasis, have been published. In NSCLC and gastric cancer, lack of TBL1XR1 expression eliminated cell migration and invasion, whereas forced TBL1XR1 expression enhanced cell migration and invasion (35, 41, 51). TBL1XR1 overexpression LUSC cells appeared to have a fusiform appearance with protuberances, which was indicative of the phenotype of mesenchyme (51). In contrast, the quantity of erratic branching structures was decreased by TBL1XR1 knockdown and some round-shaped cells which was related to an epithelial phenotype increased significantly (51). Additionally, after TBL1XR1 knockdown in gastric cancer cells, mesenchymal cell markers expression dropped while the epithelial cell indicators levels considerably rose (35). To further measure the influence of TBL1XR1 on gastric cancer metastatic and dissemination potential in animals, TBL1XR1 knockdown cells and control cells were intraperitoneally transplanted into nude mice and peritoneal nodules were fewer after inhibition of TBL1XR1 (35). In osteosarcoma cells, TBL1XR1 knockdown inhibited cell migration and invasion and miR-186-5p’s functional impacts on invasion and migration are mediated by TBL1XR1 (37). In mouse organoids of gastric cancer, TBL1XR1 knockdown reduced EMT and inhibited lung metastasis (46). Moreover, TBL1XR1 knockdown prevented CD44+ GC from promoting tube development, and animals with TBL1XR1 knockdown-transduced footpad tumors had considerably smaller popliteal lymph nodes than control mice (46). In ESCC, both *in vivo* and *in vitro*, enhanced expression of TBL1XR1 accelerated lymphangiogenesis and lymphatic metastasis (33). In summary, TBL1XR1-mediated EMT and angiogenesis cooperatively promote cancer metastasis.

### 5.5 Role of TBL1XR1 in stemness

Cancer stem cells are essential for the regulation of tumorigenesis, tumor growth, chemoresistance, and cancer recurrence. TBL1XR1 overexpression increased spheroid formation and upregulated Sox2 and CD44 expression in gastric cancer cells (46). Additionally, human gastric cancers that showed elevated TBL1XR1 expression had higher levels of CD44 than those had reduced TBL1XR1 expression, indicating a strong link between TBL1XR1 and CD44 (46). Osteosarcoma cells that have been transduced with TBL1XR1 produced greater numbers of spheres and had a higher percentage of SP+cells which processed stem cell-like characteristics (48). Meanwhile, osteosarcoma cells with overexpressed TBL1XR1 had higher expression of stemness markers including ABCG2, BMI1, SOX2, NANOG, and OCT4, indicating that TBL1XR1 endowed osteosarcoma cells with stem cell-like properties (48). Finally, in human osteosarcoma samples, TBL1XR1 protein levels strongly associated with mRNA expression levels of the stemness markers (48).

## 6 The molecular basis underlying the regulatory mechanisms of TBL1XR1

**Figure 3:**
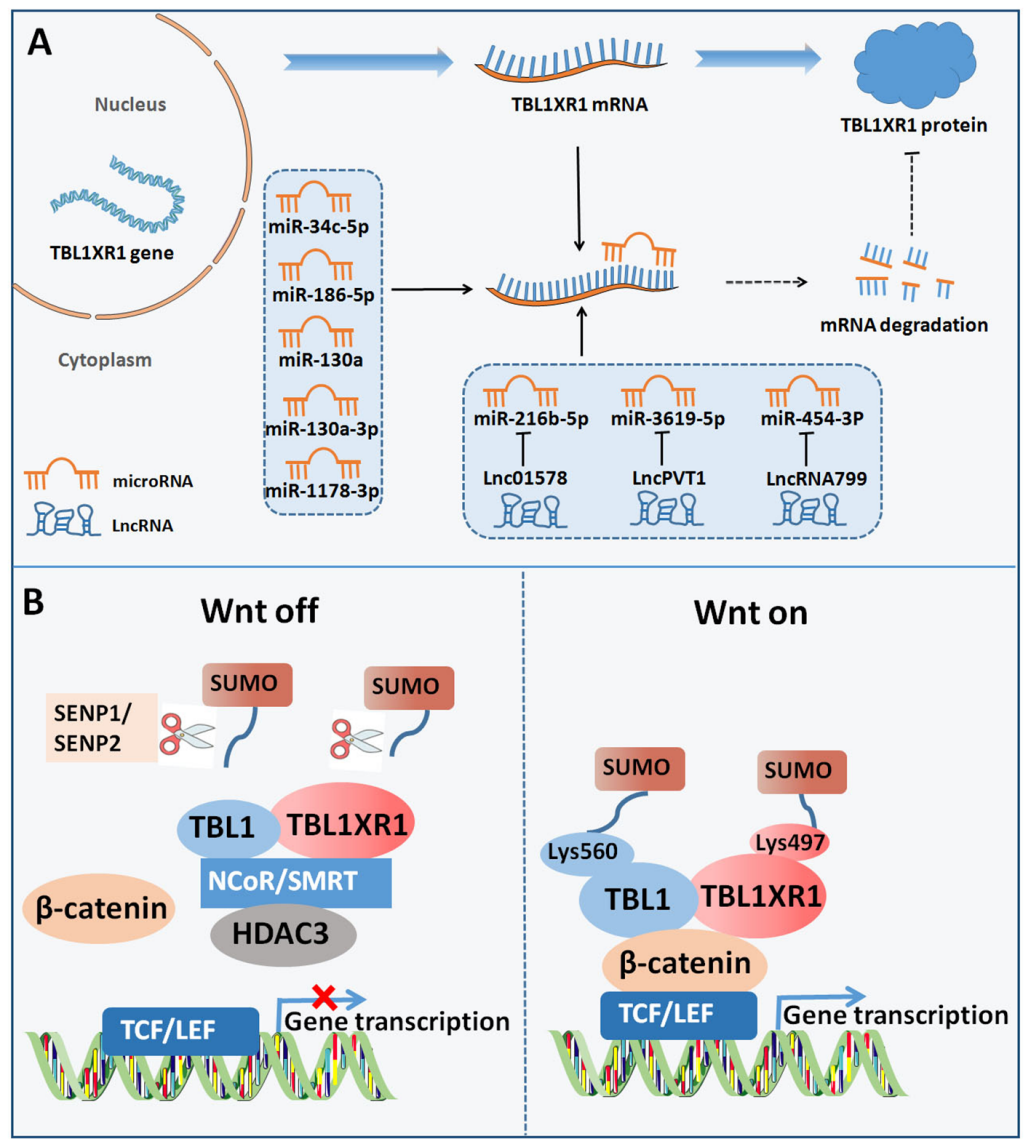
Upstream regulators of TBL1XR1. **(A)** Post-transcriptional regulation of TBL1XR1 expression via miRNAs and lncRNAs. **(B)** SUMOylation of TBL1XR1 regulated activation of Wnt-β-catenin pathway.

**Figure 4:**
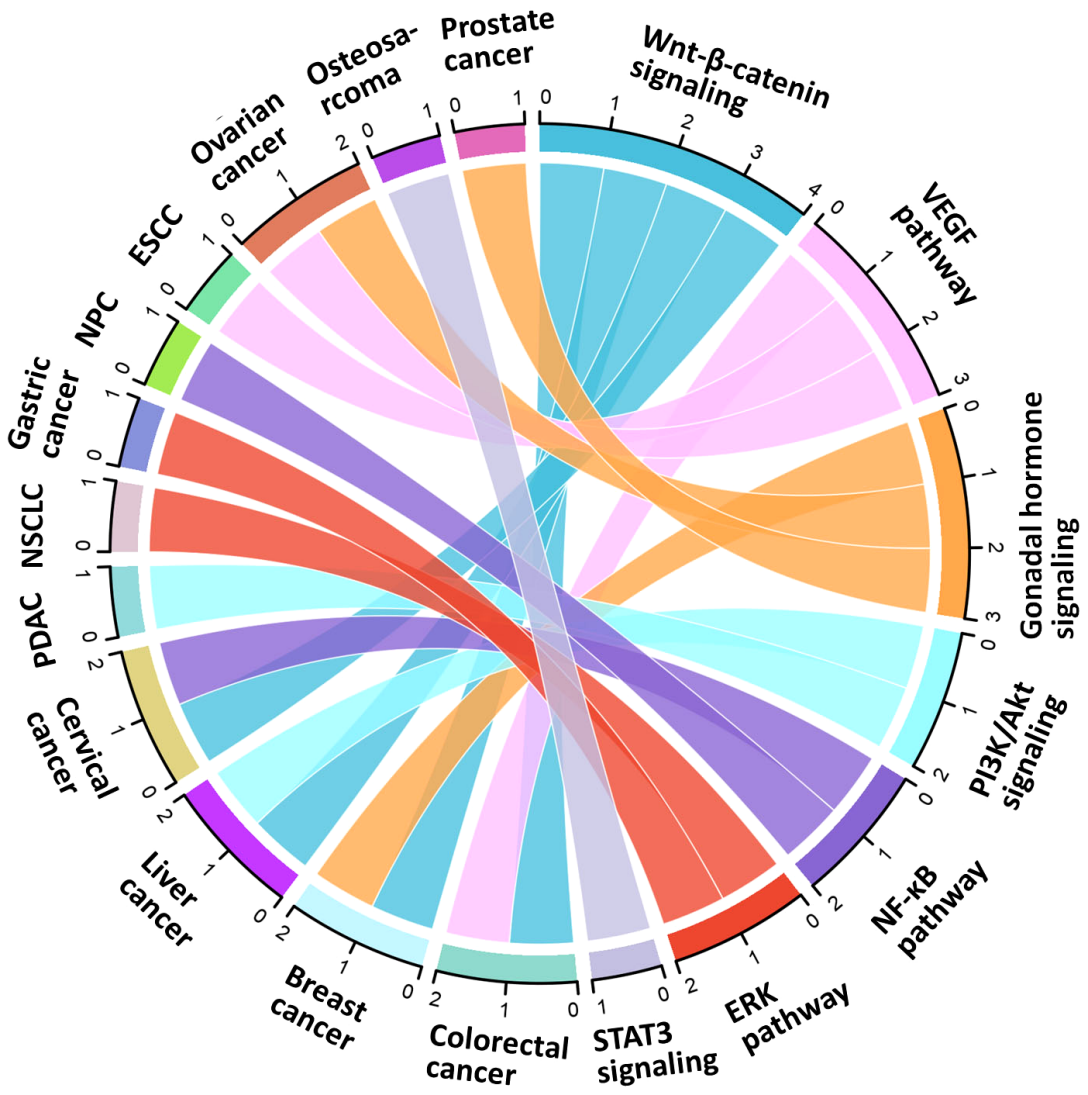
Signaling axes modulated by TBL1XR1 in cancer.

We first outlined the upstream regulators of TBL1XR1, including post-transcriptional regulation and post-translational modification, in light of the complex function and upregulation of TBL1XR1 in carcinogenesis (**Figure 3**). Then, we address more detail about the cancer pathways that TBL1XR1 modulates and further elucidate the molecular foundation for TBL1XR1’s potential as a cancer target (**Figure 4**).

### 6.1 Upstream regulators

#### 6.1.1 MiRNAs

Non-coding RNAs (ncRNAs) are essential to regulate the development and advancement of different malignancies. Targeting the 3’UTR of target genes, microRNAs (miRNAs) are a significant class of small ncRNAs that alter the expression of genes at the post-transcriptional stage (56). The emergence of miRNAs has been one of the leading factors in tumorigenesis through regulating cell growth, cell differentiation, cell metastasis, and so on (57). Considering the complex roles of TBL1XR1 in cancer, a variety of miRNAs may control the activity of various cancer cells by targeting TBL1XR1. MiR-34c-5p may largely inhibit the malignant patterns of cancer cells by down-regulating TBL1XR1 in lung cancer (58). In osteosarcoma cells, by specifically targeting TBL1XR1, miR-186-5p was downregulated and prevented cell proliferation, migration, and cell invasion (37). Furthermore, miR-130a was screened as distinctly expressed miRNAs in normal and malignant human hematopoietic stem cells. MiR-130a was overexpressed, which reduced B lymphoid differentiation and increased the number of long-term hematopoietic stem cells through targeting TBL1XR1 (59). In another study, by directly targeting and reducing TBL1XR1 expression, miR-130a-3p reduced the malignant behavior of gastric cancer cells (60). Additionally, miR-103a-3p secretion in exosome in serum of childhood pneumonia patients was downregulated and miR-103a-3p markedly reduced the immune response brought on by lipopolysaccharide through targeting TBL1XR1 (60). In hepatocellular carcinoma, through functionally inhibiting the TBL1XR1/PI3K/Akt signaling pathway, miR-1178-3p acted as a tumor suppressor (45). In conclusion, miRNAs may specifically target and suppress the expression of TBL1XR1, thereby reducing the development of cancer.

#### 6.1.2 LncRNAs

Long ncRNAs (lncRNAs) belong to ncRNAs that are greater than 200 bp. Importantly, lncRNAs participate in the epigenetic control of target genes, act as sponges for miRNAs, and regulate the occurrence of cancer by interacting with the target genes (61). Lnc01578 was reported to promote lung cancer radiation resistance through decreasing miR-216b-5p expression and elevating TBL1XR1 expression (42). Another study indicated that lncRNA plasmacytoma variant translocation 1(PVT1) increased resistance to treatment with cisplatin by upregulating TBL1XR1 via sponging miR-3619-5p (53). In addition, lncRNA799 promoted metastasis of cervical cancer via altering the production of TBL1XR1 by functioning as a relatively competitive endogenous RNA (ceRNA) for miR-454-3P (62).

#### 6.1.3 Post-translational modification

SUMOylation is commonly identified as a type of post-translational modification which requires the interaction between small ubiquitin-like modifiers (SUMOs) and substrate proteins (63, 64). Target proteins’ enzymatic activity, subcellular distribution and stability are all influenced by SUMOylation, which controls the interaction between proteins (63, 64). One research revealed that the SUMOylation of TBL1XR1 on Lysine 497 and SUMOylation of TBL1 on Lysine 560 dismissed TBL1-TBL1XR1 from the NCoR/HDAC3 corepressor complex, in favor of the enhancement of β-catenin-mediated transcription (65). On the contrary, SUMO-specific protease I (SENP1) inhibited β-catenin-mediated transcription by reducing the development of the TBL1-TBL1XR1–catenin complex (65). Functionally, SUMOylation of TBL1XR1 enhances the tumorigenic growth of SW480 cells both *in vitro* and *in vivo (* 65). In bladder cancer, SUMO-specific protease 2 (SENP2) decreased MMP13 expression through de-SUMOylation of TBL1XR1, thereby inhibiting nuclear translocation of β-catenin (66). In summary, TBL1XR1 was regulated by SUMOylation at post-translational level to modulate the function of Wnt-β-catenin pathway.

Expect for SUMOylation, TBL1XR1 regulation was also tightly controlled by phosphorylation. Upon stimulation with retinoic acid or estrogen, PKC directly phosphorylated TBL1XR1 at the Ser123 site on regulated promoters *in vivo (* 67). However, phospho-TBL1XR1 was rapidly poly-ubiquitylated and degraded (67). Poly-ubiquitylated, phosphorylated TBL1XR1 always accumulated in the nuclear fraction, allowing for exerting its function.

### 6.2 TBL1XR1-modulated cancer-associated signaling pathways

#### 6.2.1 Wnt-β-catenin signaling

Excess Wnt signalling activation enhances oncogenesis by accelerating β-catenin nuclear accumulation to trigger the activation of downstream target genes (68). TBL1XR1 was reported to promote cancer metastasis via activating Wnt-β-catenin signaling. TBL1-TBL1XR1 were necessary for gene transcription which was controlled by Wnt-β-catenin pathway and TBL1-TBL1XR1 and β-catenin were both required for enrollment to Wnt target-gene promoters (9). Suppression of TBL1-TBL1XR1 strongly inhibited Wnt-induced gene expression and several malignant properties, indicating the involvement of TBL1XR1 in Wnt signalling (9). The SUMOylation of TBL1-TBL1XR1 increased the activity of Wnt signaling pathway and finally promoted the colon cancer cells tumorigenic growth (65). In breast cancer cells, Wnt/β-catenin target genes (MYC, CD44, CYR61, Snail, AXIN2, RUNX2, LEF1, NRCAM) were significantly increased after TBL1XR1 overexpression, but decreased after TBL1XR1 knockdown (44). Intriguingly, TBL1XR1 increased the expression of MYC and cyclin D1 by directly interacting with the promoters of genes (44). Moreover, the regulation of TBL1XR1 on cyclin D1 and TBL1XR1-induced proliferation was eliminated by the suppression of Wnt/β-catenin signaling (44). In human breast cancer tissues, Cyclin D1 and β-catenin expression were positively linked with TBL1XR1 expression (44). By blocking the Wnt/β-catenin pathway, the stimulatory effect of TBL1XR1 on EMT of hepatocellular carcinoma may be eliminated (39). Another study in cervical cancer revealed that TBL1XR1 directly bound to the snail promoter and the Twist promoter (49). TCF4-RNAi treatment resulted in more pronounced deregulations of fibronectin, Twist, vimentin, as well as Snail in TBL1XR1-overexpressing cells (49). By controlling the expression of Snail and Twist, TBL1XR1 could influence EMT to cause cervical cancer cells to metastasize via the Wnt/-catenin pathways.

#### 6.2.2 PI3K/Akt signaling

The PI3K/Akt pathway exerts a necessary role in many regular cell processes such as cell proliferation, cell apoptosis and cell cycle. It is aberrantly activated in numerous cancers, contributing to the progression and occurrence of tumors (69, 70). In TBL1XR1 knockdown PDAC cells, the activation of PI3K/Akt pathway was obviously decreased and TBL1XR1 deletion-induced suppression of proliferation was modulated through the PI3K/AKT pathway (50). Additionally, treatment with LY294002, one of the PI3K/Akt inhibitors, abolished the effect of TBL1XR1-mediated proliferation increase and apoptosis suppression (50). In hepatocellular carcinoma, TBL1XR1 mediated the PI3K/Akt regulation by miR-1178-3p (45).

#### 6.2.3 ERK pathway

Dysregulation of the ERK pathway is closely related with the oncogenesis of human cancers by facilitating tumor proliferation, invasion, metastasis, and angiogenesis which are appealing targets for anti-cancer therapeutics (71). In both the gastric cancer tissues and cancer cell lines, TBL1XR1 and pERK1/2 expression levels showed a positive correlation (35). By stimulating the ERK1/2 signaling pathway, TBL1XR1 controls EMT process, cell migration, cell invasion, and cell proliferation of gastric cancer cells (35). Furthermore, the β-catenin/MMP7/EGFR signaling pathway was essential for mediating the activation of ERK1/2 pathway caused by TBL1XR1 overexpression (35). Phosphorylation of ERK1/2 was observed following TBL1XR1 knockdown and this effect was dependent on PI3K/Akt pathway in gastric cancer stem cells (46). TBL1XR1 governed Akt and ERK signaling pathways activation that were mediated by c-Met, and it was necessary for the proliferation and chemoresistance of NSCLC cells (41).

#### 6.2.4 NF-κB pathway

NF-κB pathway has attracted increasing attention in cancer research and its aberrant activation is frequently existed in various tumors (72). In NPC cells, the mRNA levels of a large number of downstream target genes of NF-κB as well as the ability of NF-κB to bind DNAs were associated with TBL1XR1 protein levels (34). The NF-κB signaling pathway was activated by TBL1XR1, subsequently conferring the NPC cells with anti-apoptotic features (34). In another study, TBL1XR1 overexpression dramatically improved, but TBL1XR1 knockdown strongly decreased, the NF-κB luciferase reporters activity (49). Furthermore, TBL1XR1-induced aggressiveness was abolished when an IkBα dominant-negative mutant was transfected into TBL1XR1 overexpression cervical cancer cells (49). Thus, TBL1XR1 induced cervical cancer cells EMT partially by regulating the NF-κB pathway.

#### 6.2.5 VEGF signaling pathway

VEGF-C, widely known as a lymphangiogenic growth factor, mediates cancer-mediated lymphangiogenesis via inducing lymphatic endothelial cells proliferation and migration and meanwhile stimulating blood vessel penetration (73). According to Liping Liu et al., TBL1XR1 increased VEGF-C mRNA levels in ESCC cells and controlled the luciferase activity that was activated by the VEGF-C promoter (33). Further study demonstrated that TBL1XR1 bound to two regions of nucleotides (−1162 to −1019 and +186 to +347) in the VEGF-C promoter (33). Functionally, both AKT and ERK activation as well as TBL1XR1-induced lymphangiogenesis were dependent on VEGF-C in ESCC (33). In ESCC tissues, TBL1XR1 expression levels were significantly linked with VEGF-C expression, indicating the role of TBL1XR1 in ESCC angiogenesis (33). TBL1XR1 silencing in ovarian cancer cells decreased VEGF-C expression and a strong association between VEGF-C and TBL1XR1 was revealed in ovarian cancer tissues (32). In colorectal cancer, TBL1XR1 also mediated the modulation of VEGF-C, thus mediating the process of lymph node metastasis (74).

#### 6.2.6 Gonadal hormone signaling

Recently, more and more researches have revealed that a major risk factor for the genesis and growth of gonadal hormone-related malignancies is aberrant gonadotropin signaling activation. ER was crucial in the initiation and spread of breast and ovarian cancer while AR exerted important roles in prostate cancer. According to reports, TBL1XR1 inhibited transcriptional activation controlled by ER in breast and ovarian cancer cell lines by acting as an ER corepressor (36). But in these cells, TBL1XR1 had no impact on AR-mediated transcriptional activation (36). In prostate cancer cells, TBL1XR1 played an essential role in AR-mediated transcription and there was physical interaction between TBL1XR1 and AR (38). More importantly, posttranslational regulation including amino acid phosphorylation and ubiquitination were important for TBL1XR1 action on AR-mediated transcription (38).

#### 6.2.7 STAT3 signaling

The persistent activation of STAT3 maintains a pro-carcinogenic microenvironment and mediates tumour-promoting processes (75, 76). The activity of the STAT3 luciferase reporter was greatly increased when TBL1XR1 was overexpressed, while it was significantly decreased when TBL1XR1 was inhibited (48). Additionally, STAT3 target genes levels (SOCS3, VEGFA, IL6, IL8, GDNF, ABCB1, TWIST1, BCL2, BCL3, BCL2L1, XIAP) were markedly elevated in the cells with TBL1XR1 transfection, but were decreased in TBL1XR1 knockdown cells (48). More importantly, the influence of TBL1XR1 on tumor development of osteosarcoma were mediated by stimulation of the STAT3 signaling pathway (48).

## 7 Conclusions and future perspectives

**Figure 5:**
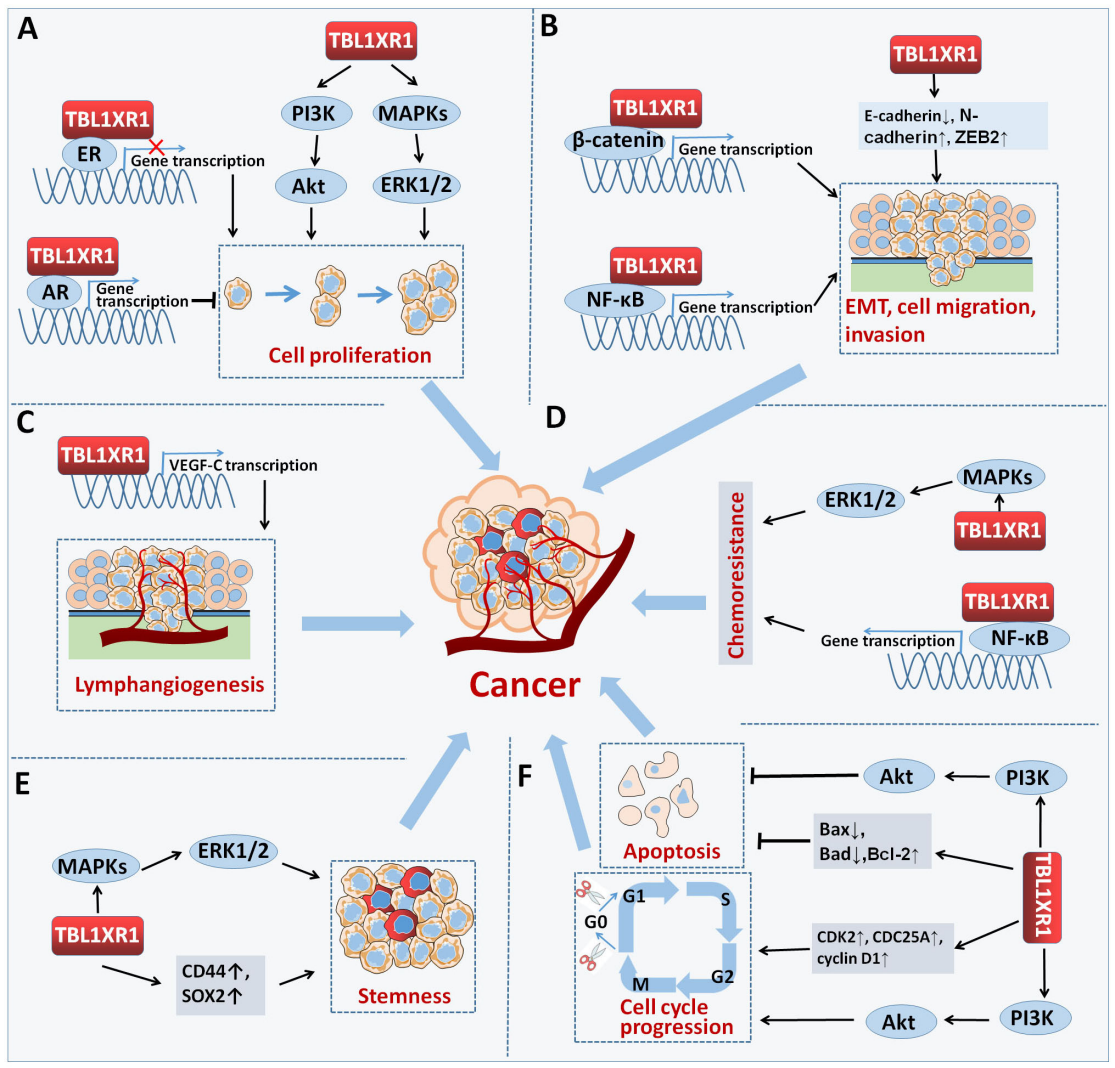
TBL1XR1 in cancer. TBL1XR1 is involved in multiple processes of cancer occurrence and development. **(A)** Promoting cell proliferation. **(B)** Facilitating EMT, cell migration and invasion. **(C)** Promoting lymphangiogenesis. **(D)** Accelerating chemoresistance of cancer cell. **(E)** Facilitating stemness. **(F)** Inhibiting cell apoptosis and inducing cell cycle arrest.

TBL1XR1 is an intriguing target for cancer treatment due to the ongoing discoveries about its role in carcinogenesis. It is evident that TBL1XR1 can be used as a biomarker due to its clinical significance in both disease diagnosis and patient prognosis (**Table 1**). TBL1XR1 is overexpressed in most cancers, and it is a driver of oncogenesis stimulating cancer cell proliferation, exerting anti-apoptotic effects, maintaining stemness, promoting EMT process, and increasing resistance to traditional cancer treatment (**Figure 5**). Now, we builds up a generalized overview of TBL1XR1 biology in the context of cancer. Nevertheless, further extensive studies are required to divulge small molecular compounds or natural compounds targeting TBL1XR1. Improved knowledge of TBL1XR1 will be helpful for deep understanding of novel molecular mechanisms of tumorigenesis and even providing clinical translation for anticancer therapy in human cancers.

## Author contributions

RD: Funding acquisition, Writing – original draft. KL: Funding acquisition, Software, Writing – review & editing. KG: Software, Writing – review & editing. ZC: Software, Writing – review & editing, Visualization. XZ: Software, Visualization, Writing – review & editing. LH: Writing – review & editing, Conceptualization, Supervision. HB: Conceptualization, Supervision, Writing – review & editing, Funding acquisition.
